# Electrospun nanofibers: A nanotechnological approach for drug delivery and dissolution optimization in poorly water-soluble drugs

**DOI:** 10.5599/admet.844

**Published:** 2020-07-05

**Authors:** Luis Castillo-Henríquez, Rolando Vargas-Zúñiga, Jorge Pacheco-Molina, Jose Vega-Baudrit

**Affiliations:** 1 Physical Chemistry Laboratory, Faculty of Pharmacy, University of Costa Rica, 11501-2060, San José, Costa Rica; 2 National Laboratory of Nanotechnology (LANOTEC), National Center for High Technology (CeNAT), 1174-1200, San José, Costa Rica; 3 Laboratory of Pharmaceutical Technology, Faculty of Pharmacy, University of Costa Rica, 11501-2060, San José, Costa Rica; 4 Laboratory of Polymers (POLIUNA), Chemistry School, National University of Costa Rica, 86-3000, Heredia, Costa Rica

**Keywords:** Electrospinning, Drug development, Drug loading, Drug release, Nanotechnology, Polymers, Solubility

## Abstract

Electrospinning is a novel and sophisticated technique for the production of nanofibers with high surface area, extreme porous structure, small pore size, and surface morphologies that make them suitable for biomedical and bioengineering applications, which can provide solutions to current drug delivery issues of poorly water-soluble drugs. Electrospun nanofibers can be obtained through different methods asides from the conventional one, such as coaxial, multi-jet, side by side, emulsion, and melt electrospinning. In general, the application of an electric potential to a polymer solution causes a charged liquid jet that moves downfield to an oppositely charged collector, where the nanofibers are deposited. Plenty of polymers that differ in their origin, degradation character and water affinity are used during the process. Physicochemical properties of the drug, polymer(s), and solvent systems need to be addressed to guarantee successful manufacturing. Therefore, this review summarizes the recent progress in electrospun nanofibers for their use as a nanotechnological tool for dissolution optimization and drug delivery systems for poorly water-soluble drugs.

## Introduction

Nanofibers can be considered as nanomaterials based on their diameter, because they geometrically fall into the category of one-dimensional nanoscale elements, such as nanotubes and nanorods. However, their highly flexible nature makes them similar to two-dimensional elements and others like globular molecules as well. In addition to that, they can be viewed as nanostructured materials when filled with nanoparticles in order to form a composite. Therefore, nanofibers possess characteristics such as high surface area, extreme porous structure, small pore size and surface morphologies that make them suitable for drug development and biomedical applications, providing sophisticated and novel solutions to current drug delivery inconveniences [1-3].

Electrospun nanofibers have been the target of different applications, due to their structure and physicochemical properties. These types of nanofibers have been studied by Schaub *et al.* based on their regenerative potential in spinal cord injuries [4, 5]. According to Sundara *et al.*, their 3D architecture makes them very similar to the skin extracellular matrix structure, which allows their use as scaffolds for skin tissue engineering [6]. In addition to that, Miguel *et al.* have worked with electrospun nanofibers loaded with bioactive molecules for improving wound healing [7]. Another application, sustained release, has been evaluated by Chou *et al.* for hydrophilic small molecule drugs [8]. Recently, Woon and Sun have developed a charged poly(vinylidene fluoride) (PVDF) nanofiber filter to overcome novel coronavirus (SARS-CoV-2) capture limitations exhibited by N95 and N98 masks. This technology effectively traps virus particles at 100 nm, which reduces the risk of acquiring COVID-19. Their work might set new technologies for protective clothing from biological agents as well [9]. Some other uses that have been given to electrospun nanofibers within the biomedical and pharmaceutical industries are water filtration, cosmetic masks, and nanosensors [10, 11].

It is worth mentioning that a wide array of polymer types are being used in the creation of nanofibers, like synthetic, natural, biodegradable, non-degradable or a blend of them. Thanks to the nanotechnological boom, many techniques are available and employed for the manipulation of polymers and nanofibers’ preparation [12-14]. Methods can be either chemical or physical; some of these are drawing, melt blowing, template synthesis, sea-island spinning, self-assembly, phase separation and electrospinning [15-20]. Among the mentioned, electrospinning is the most employed method for the production of nanofibers as an alternative delivery strategy for poorly water-soluble drugs that are not suitable for oral administration [21, 22]. Also, it is considered as the most efficient technique since it has been recognized as a simple, versatile and low-cost process. Electrospun nanofibers are produced by an electrostatically driven jet of a solid dispersion consisting of a polymer solution. Compared to other conventional solid dispersion techniques, it can produce nanofibers with enhanced dissolution and improved physicochemical properties of active pharmaceutical ingredient’ (API) particles, which can be attributed to the amorphization of the enclosed drug [23-26].

However, among the mentioned applications of electrospun nanofibers, the most relevant is their capacity to act as drug delivery systems due to their high loading capacity, faster dissolution kinetics, APIs simultaneous delivery, and encapsulation. These outstanding properties make them attractive for the industry [27-30]. Therefore, the present review focuses on the different electrospinning methods and materials employed for obtaining nanofibers, as well as recent and relevant investigations that have improved the delivery and dissolution performance of poorly water-soluble drugs, including smart nanofibers.

## Historical context

In the 17^th^ century, William Gilbert was the first in documenting research with high voltage direct current applied to a solution. He observed a cone shape when a drop of water was close to electrically charged amber [31]. By the end of the 1800s, many papers had been released regarding the use of ‘electrical spinning’, using materials such as shellac, beeswax, and others, whilst in the early 1900s, the electrical charge was used mainly to spray liquids, as established by Cooley and Morton in their patents about electrical methods of dispersing fluids [32, 33].

Nevertheless, the first electrospinning patent ‘Process and apparatus for preparing artificial threads’ came in 1934 by Formhals, where he described an experimental setup characterized by a collecting device, drum wheel and a plate for the production of polymer filaments using electrostatic force, which later was going to be a novel technique in nanotechnology [34]. However, it was not until 1974 when Taniguchi in his publication ‘On the basic concept of nanotechnology’, that this scientific field was referenced for the first time [35]. Nanotechnology is conceived in its simplest definition as the science and technology that takes place on the nanoscale (1-100 nm). Precisely, due to the previous, it has been possible to obtain novel materials and structures that are integrated into larger systems improving not only chemical, physical and biological properties, but also different kinds of processes [36, 37].

Although electrospinning as a polymer-processing technology has been known for more than 80 years, it was not until 1994 when significant studies explored the production of nanofibers through this technique. By that year, the term ‘electrospinning’ was adopted among the scientific community thanks to the work of different researchers including Doshi, Reneker, and Chun, who demonstrated that many organic polymers can be electrospun into nanofibers [38, 39]. Since then, significant effort has been done for the study and employment of this nanofiber fabrication technique.

## Electrospinning techniques

It is well known that electrospinning has its origin in ‘electrospraying’, wherein both cases the application of an electric potential to a polymer solution causes a charged liquid jet that moves downfield to an oppositely charged collector [40, 41]. Nevertheless, electrospraying is performed using a low viscosity liquid which due to the surface tension produces instability in the jet, resulting in the formation of polymer droplets instead of nanofibers, as occurs in electrospinning when working with polymer solutions of higher viscosity. The process can be done under room temperature unless it is necessary a heat source to keep the polymer molten [41, 42].

**Figure 1.**
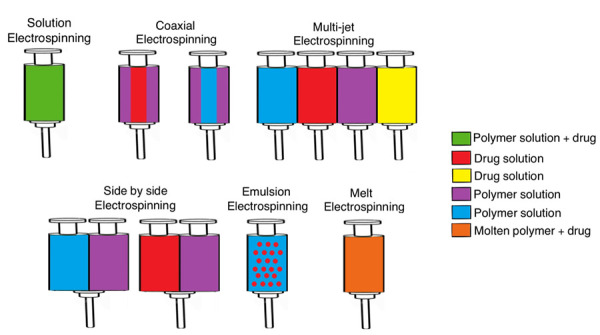
Electrospinning techniques.

In addition to that, nanofibers properties will depend on the type of polymer used and the method of manufacture, since there have been some modifications to the conventional electrospinning technique. The methods can be classified as needle or needleless electrospinning, where the latter is represented by roller and wire electrospinning, which offer some advantages over needle methods like the lack of clogging [43, 44]. However, those advantages are not as significant as the differences within needle systems, thus this review will focus on needle techniques presented in Figure 1. A single needle set-up, like solution electrospinning, is mostly used for research purposes, while multiple systems such as coaxial, multi-jet, side by side, emulsion, and melt electrospinning have been designed to improve nanofibers manufacturing [45, 46].

### Solution electrospinning

**Figure 2.**
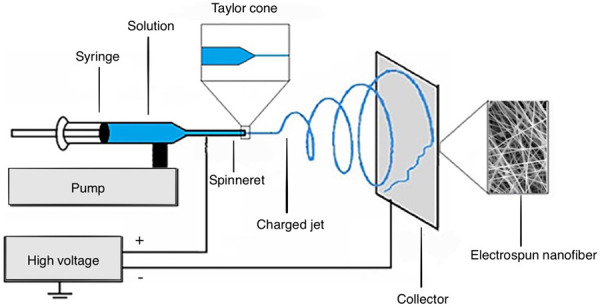
Electrospinning basic set-up design. Adapted with permission from Y. Li *et al.* Electrospinning in Tissue Engineering. In: A. Haider, S. Haider. Electrospinning: Material, Techniques, and Biomedical Applications p.117-139. Copyright (2016) IntechOpen [51].

Also known as co-electrospinning or blending electrospinning, it is conceived as a solid dispersion method that involves the dissolution of a drug in a polymer solution using a suitable solvent. It requires simpler equipment than other fiber manufacturing techniques, but it can create a great variety of products for different applications [47, 48]. The usual set up design showed in Figure 2 consists of a feeding unit with a spinneret, typically a syringe controlled by a pump that transports the polymer solution. Some novel spinnerets recently used by several research groups for improving the nanofiber fabrication process are rotating disk, plucked string, plated edge, and multiple rings. There is also necessary a high-voltage supplier connected to the spinneret and the collector, charging them oppositely [49-51].

Regarding the process, the polymer solution is held by its surface tension at the end of the spinneret or capillary tip. Once the solution is subjected to high voltage by a direct current power supply, the droplet at the tip elongates to develop a conical form known as ‘Taylor Cone’ [52]. When the electric field or electrostatic repelling force between the spinneret and the collector reaches a critical voltage, it overcomes the surface tension of the polymer solution, resulting in the ejection of a continuously charged jet from the tip of the Taylor cone [53-55].

As it travels to the rotating or stationary collector two main events are worth highlighting. In the first place, the solvent evaporates but not completely, which represents a challenge for research groups due to solvent’s toxicity. On the other hand, the fine filament travels following a chaotic trajectory in a whip-like motion, known as bending instability. When it reaches the collector the continuous filament accumulates or deposits to form the nanofiber [56-60].

Tan *et al.* employed this technique to fabricate vascular grafts made of poly(ɛ-caprolactone) (PCL), gelatin, and poly(vinyl alcohol) (PVA). The obtained nanofibers rapidly degradated *in vivo,* also possessed appropriate properties for use in artery replacement, and offered scaffolds with high porosity enhancing cell proliferation and infiltration [61]. In another approach, Ahmed *et al.* reported the creation of PCL /cellulose nanofibers for the use as biosensor strips [62].

### Coaxial electrospinning

Coaxial electrospinning presents changes regarding the method described in solution electrospinning. In this multiple needle system, two polymer solutions or a polymer and a drug solution with different properties regarding solubility and hydrophilicity, are loaded into individual feeding units to simultaneously electrospin from coaxial spinnerets. Since the last ones share an axis, one solution is injected through the inner needle into the concentric outer one at the capillary tip, resulting in a continuous core-shell nanofiber. In addition to that, the principle can be applied to triaxial systems as well, where the electrospun nanofiber will contain a core, a middle layer, and sheath [63-65].

The previously described process allows confining APIs inside the core and controlling drug delivery mainly through diffusion rather than desorption of the surface [66]. Moreover, coaxial electrospinning using hydrophilic and hydrophobic polymers within the composition provides a rich combination of their properties in a single engineered nanofiber while each component maintains its identity. This type of electrospun nanofiber leads to better control of drug diffusion to the medium [67-69].

Wang *et al.* provided an approach for the design of hypromellose (HPMC)-based hydrophilic composites fabricated through coaxial electrospinning. The core fluid of the electrospun nanofibers was prepared from an electrospinnable solution containing 13% (w/v) of HPMC, 2% (w/v) of ferulic acid and 3% (w/v) of poly(ethylene glycol) (PEG) in a 1:1 ethanol/dichloromethane (DCM) mixture, while ethanol was used as the sheat solvent for the process. The performance of the electrospun nanofibers in the dissolution test revealed to be 10 times faster than casting hydrophilic composites. Therefore, this approach can be useful as a means to obtain carriers for the delivery of poorly water-soluble drugs [70]. Another research published by Wu *et al.* proposes an alternative approach. In this case, the sheath layer consisted of a solution of poly(vinylpyrrolidone) K60 (PVP) and citric acid, while the core was composed of a PEG 6000 and sodium hydroxide co-dissolving solution [71].

Like in solution electrospinning, the application of a high voltage results in the formation of a Taylor cone. However, in this case, it is compounded and formed by the core solution surrounded by the sheath polymer solution [72, 73]. This method can solve the problem of those highly unstable substances of biomedical interest like enzymes, which can’t be electrospun due to their molecular weight or solubility properties. Furthermore, when these compounds are loaded into the core the sheath structure protects them from the reactive environment [74].

### Multi-jet electrospinning

A multiple spinnerets or nozzles set-up design is readily used for increasing the production rate of electrospun nanofibers due to its simplicity. This method can be classified into three categories: a) single spinneret with multiple jets, b) multiple spinnerets and each one with a single jet, and c) multiple spinnerets with multiple jets per each. Therefore, a mass production increase of nanofibers can be achieved by increasing not only the nozzles but also the tips or holes [75-77].

Multi-jet electrospinning possesses a very similar process compared to the conventional method, where a continuously charged jet trajectory is defined by the electrical field. However, in this system, the jet direction towards the collector is greatly influenced by Coulomb forces caused by neighboring jets. Precisely, one of the major issues of this method is a jet deviation that is exerted by the interaction of electrostatic forces between them, which provokes defects in the nanofibers [75, 78, 79].

In order to reduce jet repulsion, many approaches have been performed such as increasing the applied voltage, different spinnerets arrangements, and adjusting the distance between them. Regarding that, Liu *et al.* proposed placing an auxiliary grounded electrode to solve the problem, which improves the production rate as well [80]. Later work from Liu’s group evaluated the influence of solution properties including dielectric constant, polarity, conductivity and surface tension on multi-jet electrospinning when using an auxiliary electrode. This time it was found out that solutions with higher dielectric constant (32.2-78.6) and larger surface tension (31.8-41.29 mN/m) are most likely to produce 2-6 jets with short stable length (1.7-6.9 mm) under the influence of low voltage (5.03-7.13 kV) [81].

### Side by side electrospinning

Sometimes it is necessary to combine blends of polymers using one solvent or different polymer solutions for nanofiber production through electrospinning. However, it is not always possible to manufacture nanofibers this way, since both polymers are required to be thermodynamically miscible when dissolving them in the same solvent. In addition, for polymer solution mixtures, polymer-polymer, polymer-solvent and solvent-solvent interactions must be analyzed [82, 83].

Thus, side by side electrospinning introduces itself as a means for overcoming these difficulties. This method focuses on controlling viscosity and conductivity of two polymer solutions that do not get into physical contact until they reach the tip of the spinneret, where they are electrospun simultaneously. Therefore, the resulting nanofiber consists of a bicomponent system with good mechanical strength and great thermal stability. In addition to that, it has different properties on both sides, based on the corresponding component placed on each [84, 85].

Yu *et al.* have employed this technique for the fabrication of Janus fibers using a teflon-coated parallel spinneret, which allowed the formation of a Janus Taylor cone for obtaining high quality integrated Janus structures. The electrospun nanofibers had one side composed of PVP K60 and ketoprofen, while the other consisted of ethyl cellulose (EC) and the drug as well. The mentioned biphasic design provided a fast dissolution on PVP K60 side and a sustained release of the remaining drug on the EC side [86]. Later, they produced high quality PVP K60/shellac Janus nanofibers using this electrospinning method, but with the modification of using a structured spinneret comprising two eccentric needles nested into a third one [87].

### Emulsion electrospinning

Proposed by Xu *et al.*, it has been developing recently as an alternative technique of electrospinning [88]. This novel method allows processing emulsions to encapsulate either hydrophilic or hydrophobic APIs for the manufacturing of core-shell nanofibers, using a single spinneret. The process has been found to enhance encapsulation in the core and to provide better drug stability and bioavailability relevant for the development of advanced drug delivery systems. The release mechanism is mainly controlled by diffusion and enzymatic degradation of the solidified polymers [89-91].

For this purpose, either water-in-oil or oil-in-water emulsions can be electrospun [89]. During the process, the solvent molecules closer to the polymeric jet surface evaporate at a higher rate, which causes a viscosity increase in the outer layer. Then, emulsion droplets are subjected to the electrical field that induces them to condense and stretch into elliptical shapes, but also several other forces have an influence on the charged jet, like Coulomb’s and electrostatic forces, which provoke droplet expansion. On the other hand, viscoelastic forces and surface tension control droplet contraction to reduce the interface between the air and the polymeric jet. Finally, when the jet reaches the collector, the solvent has almost completely evaporated [92, 93].

Hu *et al.* investigated the influence of Span 80, sodium dodecyl sulfate (SDS) and poly(ethylene oxide)-poly(propylene oxide)-poly(ethylene oxide) triblock copolymer towards the morphological properties of an electrospun poly (ɛ-caprolactone) /bovine serum albumin nanofibers. It was found that a 0.4% (w/v) SDS emulsion produced the most uniform electrospun nanofibers through emulsion electrospinning [94]. In another research, Moydeen *et al.* employed the technique for the production of core-shell PVA/dextran sulfate nanofibers loaded with ciprofloxacin, which demonstrated to improve the sustained release of the drug compared to the nanofibers obtained by co-electrospinning [95].

### Melt electrospinning

This technique is considered one of the most relevant variations of the conventional electrospinning method because instead of using a polymer solution it employs molten polymers for achieving nanofiber production [96, 97]. Although molten polymer viscosity will tend to be greater compared to a polymer solution, it is possible to use some other substances as auxiliaries like plasticizers in order to reduce this parameter. When the jet arrives at the collector the solidification process will not be caused by drying or evaporation of a solvent but it will be produced by molten polymer cooling [98, 99].

Melt electrospinning stands out for being considered as a green technology in nanofibers manufacturing, which allows exploring new approaches regarding biomedical and bioengineering applications without the restrictions of using solvents, and related issues like incomplete elimination and their toxicity [100, 101]. In addition to that, it is a promising method for industrial production and commercialization, since it not only increases the production rate of nanofibers but also gets rid of manufacturing costs represented by the use of expensive solvents [102, 103].

Larrondo and Manley were the pioneers of this method three decades ago when they tried to produce nanofibers from molten polypropylene (PP) but ended obtaining fibers with diameters greater than 50 μm due to high melt viscosity of the polymer [104-106]. Despite that, the technique hadn’t been exploited to a great extent until Weimin *et al.* proposed in 2014 a variant of this method called ‘Polymer Melt Differential Electrospinning’ (PMDES) [107].

In PMDES scale-up, it was found out that producing multiple jets from one umbrella-shaped spinneret resulted in the obtention of nanofibers with an average diameter of 300 nm, with an efficiency 500 – 1000 times higher than solution electrospinning, and about 80 times higher compared to conventional melt electrospinning [107]. However, even the basic method of this technique is still under study, thus process parameters such as melt viscosity and processing temperatures still need special attention and evaluation [108].

## Polymers and solvents

Among the most frequently used materials in nanotechnology, polymers are actively employed in this field for the production of nanofibers by electrospinning. A wide variety of them, from natural and synthetic origins, have been electrospun under different required manufacturing conditions. One can also use polymer blends to obtain nanofibers with different structures such as core-shell, ribbon-like, porous, and aligned. Therefore, polymer properties and functionalities provide nanofibers with desirable characteristics and performance to fulfill the general demands for specialized applications [109, 110].

Properties such as hydrophilic and hydrophobic nature of the raw material need to be addressed since they have a major effect on drug release from polymeric nanofiber matrix. Hydrophobic polymers provide limited diffusion of aqueous solutions into the matrix due to their restricted contact angle allowing for long-term release, while hydrophilic polymers cause the diffusion of the API to the medium by their swelling mechanism and their dissolution [111]. Furthermore, polymer crystallinity plays an important role, since amorphous regions provide better access to water molecules compared to crystalline regions. Thus, highly crystalline polymers exhibit a slower release of drugs [112].

The most popular synthetic polymers that have been electrospun are hydroxypropylmethyl cellulose (HPMC), PEO, PVP, sodium carboxymethyl cellulose (NaCMC), PVA, alginates, dextran, PCL, poly(lactic acid) (PLA), poly(glycolic acid) (PGA), poly(lactic acid-*co*-glycolic acid) (PLGA), and poly(lactide-*co*-ε-caprolactone) (PLCL). Regarding the natural raw materials, the use of chitosan, gelatin, collagen, chitin, fibrinogen, and silk fibroin have been reported [113, 114]. The aforementioned polymers are extensively used in biomedical and bioengineering due to their advantages in manufacturing such as stable mechanical properties, good biocompatibility for some applications such as scaffolds, and biodegradable behavior for drug release [115].

Despite the exceptional properties of polymers and the outstanding advances in nanotechnological manufacturing, not all of them are feasible for use in electrospinning. Moreover, it is required a solvent with the capacity of dissolving both, drug and the polymer. Solubility parameters can be useful for the selection of the most adequate solvent. Therefore, non-soluble polymers like polypropylene (PP), polyethylene (PE), and poly(phenylene sulfide) (PPS) can’t be electrospun satisfactorily [116, 117].

Polymer solubility is complex due to its molecular weight, size difference compared to the solvent, viscosity of the system and changes in terms of structure. The dissolution process is considered to occur in two stages. First, solvent molecules diffuse slowly through the polymer to produce a swollen gel, where polymer-solvent interactions are expected to break intermolecular polymeric bonds. Then, when all polymer-polymer bonds are broken, a true solution is formed [118, 119]. In addition to that, polymer solubility in a system can be explained by Gibbs free energy (Δ*G*) [120]:


(1)

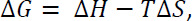



where Δ*H* is the enthalpy of the process, *T* stands for temperature and Δ*S* is the entropy. An adequate solvent is able to make polymer molecules to expand, while a poor one causes them to collapse [120]. Also, the process temperature influences the suitability of a solvent. Temperatures above Flory-Huggins temperature (*i.e.* the lowest temperature at which a polymer of infinite molecular weight is completely soluble in a determined solvent) induce polymer’s molecules to expand, which reduces Gibbs free energy of the system until it reaches a negative value where the polymer is soluble [121-123].

**Figure 3.**
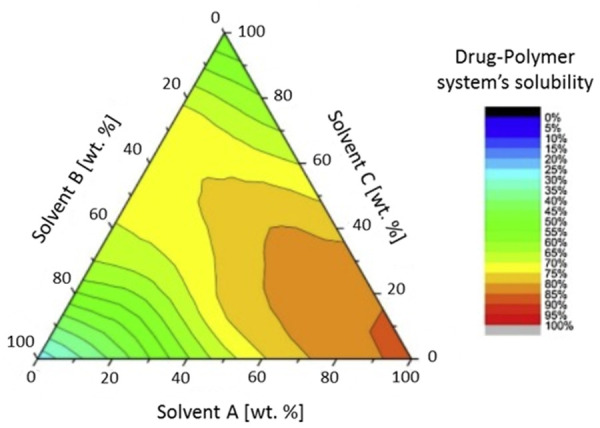
Triangular solubility diagram for a drug-polymer system in solvent mixtures. Adapted with permission from M. Knopp, *et al*. Comparative Study of Different Methods for the Prediction of Drug–Polymer Solubility. *Molecular Pharmaceutics* 12(9): 3408–3419. Copyright (2015) American Chemical Society [124].

However, in order to evaluate a solvent for a determined drug-polymer system, researchers frequently employ the triangular diagram or solubility map as the main tool for the estimation, as shown in Figure 3. Also, solvents can be classified according to their ability to dissolve the API and the polymer based on dielectric constant values within the range of interest [124, 125]. Although a polymer is soluble in a determined solvent, it does not always imply its aptitude to be electrospun for obtaining nanofibers [126].

Since different solvents display different levels of electrospinnability, choosing one entails important features to be taken into consideration besides basic parameters like dielectric constant and relative permittivity. In the first place, there must be a balance in the evaporation rate, so that it is enough to allow the fiber to maintain its integrity when arriving at the collector, but not too fast for causing hardening before it reaches the nanometer range. Furthermore, solvent viscosity and surface tension must not prevent the jet formation or allow the polymer solution to drawn freely from the spinneret [127, 128].

A polymer solution constituted by multiple solvents may present manufacturing problems, since solvents with different solubility properties can affect several factors from the polymer and the nanofibers. Polymer chain conformation, viscoelasticity, and critical minimum concentration can be influenced by the selection of the solvent [127]. On the other hand, nanofiber diameter, tensile strength, morphology and crystallinity of the loaded drug can be dramatically modified by the solvent properties [129].

Kathsee *et al.* worked with a biodegradable polymer blend composed of PLA and butylene adipate‐*co*‐terephthalate (PBAT) for the manufacturing of electrospun nanofibers. In this opportunity, they explored the effects of solution parameters such as types of binary solvents, solvent mixing ratio, and polymer blend concentration. They performed solubility tests of the PLA/PBAT blend for the selection of a suitable binary solvent system that guarantees electrospinnability, resulting in the use of a 3:2 dichloromethane (DCM)/ dimethylformamide (DMF) mixture [130]. Although the benefits of electrospinning encourage more research in this field, the non-toxic profile required for polymers and solvents has been difficult to achieve. This has represented a barrier for fast development and industrialization of the technique since more than 90% of the electrospun polymer solutions reported in the literature at present contain toxic solvents [131]. However, current trends in research have focused on developing a new generation of electrospun nanofibers with fewer toxicity problems for safe and efficacious therapeutic applications [132].

## Drug development through electrospun nanofibers

### Technological issues for the formulation of poorly water-soluble drugs

Currently, most of the drug products available do not address the growing demand for personalized therapies. Many of them face several formulation challenges due to the physicochemical properties of the APIs, which may prevent the development of a suitable pharmaceutical form that exerts an adequate therapeutic effect [133-135]. However, the rapid growth of nanotechnology intends to contribute greatly to medical and pharmaceutical sciences, providing innovative treatments that can improve patients’ lives. Thus, an increasing number of scientists, research groups, industries, and governments are investing millions of dollars for nanotechnological development [136].

It is well known that drug molecules have to be released from the matrix of the pharmaceutical dosage form and then, dissolve in order to be absorbed and to cause the intended therapeutic effect. Nevertheless, both processes are greatly dependent on the physicochemical properties of the API, and the ones from the excipients as well [137]. Consequently, Göke *et al.* state that poor water solubility is one of the major pharmaceutical challenges for drug development [138]. Amidon *et al.* classified drug molecules as a function of their aqueous solubility and intestinal permeability in the Biopharmaceutics Classification System (BCS) [139]. Classes II and IV in the BCS have poor solubility due to their complete dose not being possible to dissolve when ingested with a glass of water (250 ml) [140-142]. A great majority of new drug molecules and lead compounds are within Class II and IV, and thus present limited oral absorption, slow *in vivo* dissolution, and low bioavailability [141, 143-145].

Therefore, due to the recently increasing number of poorly water-soluble drugs, many methods have been employed for improving dissolution [146]. Electrospinning produces amorphous solid dispersions of this kind of drugs in a water-soluble matrix mainly composed of polymers than can enhance their dissolution, where that improvement is explained by the higher solubility exhibited by non-crystalline forms. In addition to that, carriers are mostly hydrophilic, and depending on the preparation method, some can cause a greater increase in the surface area than others [147, 148].

Nevertheless, drug-polymer systems are not exempt from presenting incompatibilities, not only chemical but also physical, such as a low degree of miscibility that may lead to an unstable amorphous phase [149]. On the other hand, the presence of polymorphism within the API can make harder the conversion to an amorphous structure, representing a disadvantage for the process [150]. According to Poller *et al.*, a major challenge for electrospinning is the scale-up process and this issue is currently being addressed by both academic and industrial researchers [151-153].

### Drug loading

Synthetic, natural and biologically active substances can be loaded into electrospun nanofibers and it has been widely reported by many research papers. Electrospinning provides nanofibers with a large surface area to volume ratio, which combined with the possibility of choosing an adequate solvent for solubilizing the drug of interest, makes it a technique with high loading capacity. In addition to that, the method is considered as an economic, suitable, stable and improved medium for drug loading that provides control over drug release kinetics [154-156].

Drug loading into electrospun nanofibers can be performed in different ways based on the techniques previously explained. In the first place, the drug can be dissolved directly with the polymer by the same solvent or it may require to be previously dissolved in a small amount of another one in order to be added to the polymer solution, wherein both cases the drug will end embedded in the fabricated nanofiber through co-electrospinning [154, 155]. A comparative study of curcumin-loaded PCL nanofibers by melt and solution electrospinning was carried out by Lian and Meng. They reported that the active substance did not alter the morphology of melt electrospun nanofibers, which conserved a high crystallinity and could be loaded with a large amount of curcumin in the amorphous state. However, it did cause aggregates in solution electrospinning due to its limited solubility in the solvent system composed of 3:1 DCM/ethanol, which caused jet instability during the process [157]. In another approach made by Zhu *et al*., coaxial electrospinning was employed to load flurbiprofen for obtaining electrospun PVP/PLGA core-shell nanofibers [158]. Also, for drugs that are not soluble in the same solvent as the polymer, emulsion electrospinning can directly encapsulate lipophilic or hydrophilic drugs within the polymeric matrix. Electrospinning also offers the possibility to load more than one API into the nanofiber thanks to the multi-jet variant, which is advantageous when it is needed to load lipophilic and hydrophilic drugs in the same three-dimensional structure. Moreover, according to the surface adsorption method, active substances can be incorporated after the electrospinning process by immersing nanofibers in a drug solution as well [159-161].

### Dissolution optimization

Nanotechnology has made the use of nanoparticles and nanostructures a trend for increasing the solubility of APIs, which is known to be a limiting step for bioavailability. Because of that, the dissolution test has extended its application not only to solid pharmaceutical dosage forms but to novel drug delivery systems such as electrospun nanofibers. It is well known that solubility is greatly dominated by the nature of the substance functional groups and their interactions with those of the solvent [162]. Electrospinning has allowed the production of nanofibers with poorly water-soluble drugs embedded in hydrophilic polymers, which enhances their dissolution rate [163, 164].

However, it is clearly stated in the literature that the enhanced solubility and dissolution of drugs exhibited by electrospun nanofibers is mainly due to the presence of the compound in the amorphous state. This can be explained by the fact that many APIs have higher kinetic energy in that arrangement than in the crystalline form [165]. Aside from that, a more homogenous distribution of the drug, increased wettability, lower precipitation, highly porous and specific surface area from the nanostructure also contributes to the optimization of the dissolution rate compared to the commercial pharmaceutical product or the conventional solid dispersions. These improved properties are leading to the design and development of novel formulations including buccal, transdermal and topical dosage forms [166-168].

Nazari *et al.* worked with solution electrospinning to fabricate different indomethacin buccal films made of Ethocel, HPMC and Tween 80. According to the differential scanning calorimetry (DSC) and X-ray diffraction analysis (XRD), the drug was present in the amorphous state. Dissolution performance was evaluated *in vitro* in a medium of a buffer at pH 6.8, in which it was found that the presence of Tween 80 accompanied with 5% (w/w) of HPMC caused a fast release in the first five minutes, which increased the dissolution rate of the drug in about 62% compared to the nanofiber produced with the water-insoluble polymer Ethocel [169]. Also, Szabó *et al.* produced terbinafine hydrochloride buccal films using PVA and chitosan for the electrospinning process, where the nanofibers showed fast and complete dissolution of the drug [170]. In another approach, Adeli prepared several formulations of PVP K90 electrospun nanofibers loaded with irbesartan. All the evaluated prototypes showed an improved dissolution rate, but the one that exhibited the best performance had a 3:7 drug/polymer ratio. In sink conditions, almost 97% of the drug was released in 60 minutes. Aside to that, saturation solubility increased up to 245.32 ± 1.77 μg/ml, about six times that of the pure drug (40.55 ± 1.01 μg/ml) due to the electrospun nanofibers-based solid dispersion preparation [171].

### Drug delivery and drug release

Drug delivery systems are designed for improving significantly the therapeutic efficacy and safety of a drug through controlling the targeted-site of action and the release rate. The development of this type of system involves the understanding of drug release kinetics in order to select the most appropriate compounds for the intended purpose. Therefore, controlled release is useful for modifying delivery kinetics, reducing toxicity and side effects, as well as for adjusting therapies according to patients’ convenience [172-174].

Electrospun nanofibers have been considered as suitable structures that can control spatially and temporally drug delivery of more than one drug. The first aspect can be achieved by placing the nanofiber at the targeted site by invasive or non-invasive methods to avoid systematic exposure [175-177]. Ravikumar *et al.* developed a tetrahydro curcumin transdermal patch through electrospinning solution, where the poorly water-soluble active was loaded at a concentration of 8.7% (w/w of dry polymers) into a 2:1 mix of solutions of PCL 10% (w/v) and PEG 5% (w/v) in a chloroform and acetone solvent system, obtaining nanofibers with an average diameter of 400 ± 20 nm. The cumulative % release was 95.11% at 24 hours, exhibiting first-order kinetics that matches Higuchi’s diffusion model for which this development might be feasible for once in a day transdermal drug delivery [178].

The obtained nanofibers offer different outstanding properties for hydrophobic active substances, which are explained by their nanostructured nature that improves not only physicochemical and pharmacokinetic properties but also provides protection from enzymatic or chemical degradation. This nanostructure overcomes high drug uptake limitations, exhibits efficient drug transport and has the capacity of encapsulating chemical or biological therapeutic agents during the manufacturing process [179, 180].

Paaver *et al.* successfully created supersaturated electrospun nanofibers for controlled release of piroxicam made of HPMC and 1,1,1,3,3,3-hexa-fluoro-2-propanol (HFIP) as the solvent. The dry diameter of the nanofibers was in the range of 400-600 nm based on scanning electron microscopy studies (SEM). Solid-state studies of the nanofibers carried by DSC and XRD confirmed the presence of the drug in the amorphous state, but after three months they showed a slow tendency to recrystallize into form III. Physicochemical properties of the nanofibers were strongly dependent on the concentration of HPMC, exhibited short lag-time, no initial burst release and zero-order dissolution kinetics [181].

Electrospun nanofibers are able to achieve controlled release at the targeted site by making use of several polymers, which can be biodegradable or non-degradable. According to that, drug release from non-degradable polymers is performed by diffusion, while release from degradable polymers occurs mainly by matrix erosion. However, the selection of the polymeric matrix is conditioned by the requirements of the application [182, 183]. When using natural or biodegradable polymers it is necessary to crosslink the matrices for improving the mechanical, thermochemical and structural integrity of electrospun nanofibers, which also enhances sustained and controlled released [184].

Drug release from hydrophilic polymers is possible through surface desorption, diffusion and matrix erosion due to the degradation of the nanofiber caused by enzymes. When nanofibers are constituted by this type of polymers, direct contact with an aqueous solution alters its glassy state and forms a rubbery gel, causing swelling of the polymeric matrix, which transports a drug through its network until it reaches the medium [185, 186]. For high-swelling polymers, drug release depends on the diffusion through the pores of the nanofibers, while low-swelling polymer release rate is conditioned by the swelling process itself [187, 188].

Gordon *et al.* developed research for the delivery of celecoxib lipophilic nanoparticles, which were formed by loading the poorly water-soluble drug dissolved in a volatile oil-solvent system into the electrospinning aqueous polymer solution composed of a high molecular weight PVA. The obtained hydrophilic electrospun nanofibers enabled fast dissolution and release of celecoxib nanoparticles, since the drug was present in the amorphous state and the average size of the nanoparticles was in the range of 21-93 nm [189].

Polymer, solvent and drug compatibility need to be addressed for achieving reproducible drug release and thus, drug delivery. When the electrospun nanofiber system carries multiple drugs, their affinity to the polymer as well as the interaction between them have to be considered. If any physicochemical interaction is present it may lead to problems regarding the diffusion of the drug, which will result in different release profiles [190-192]. Zhao *et al.* designed an implantable tissue-engineered scaffold through electrospinning made of a self-coated interfacial layer developed between an inorganic and an organic matrix for time-programmed multi-drug release. The scaffold’s design propitiated the absence of interactions; the inside-located drug was ibuprofen, which experienced short-term release for 30 days, while doxorubicin was loaded outside the interfacial layer and showed a sustained long-term release during 90 days [193].

Also, as stated by Haider *et al.*, the chosen electrospinning method and the process itself allow controlling the release kinetics through some critical parameters such as matrix properties, nanofiber diameter, porosity and of course their morphology. However, if the different drugs are loaded by the same electrospinning method, the release will depend on each diffusion coefficient [194]. Thus, different techniques are associated with different rates of release. When drugs are loaded through physical or surface adsorption into the nanofibers this will exhibit short diffusion times, while methods like coaxial electrospinning provide controlled and sustained profiles [195].

However, recent research is focusing on developing electrospun nanofibers that are responsive to certain stimuli or feedback factors that start the controlled release of the drug at the targeted site. These novel drug delivery systems are called smart electrospun nanofibers [196, 197].

### Smart electrospun nanofibers

Although these systems have experienced considerable progress in the past years, all of them face the same challenges regarding the manufacturing process, quality control and cytotoxicity, which imply a great barrier to faster development [196]. However, it seems that electrospinning can offer a solution to many of the common problems through the selection of a suitable carrier based on the therapeutic goal, material safety profile, drug physicochemical properties, and the route of administration. As presented on Table 1, several types of electrospun smart nanofibers have been developed that respond to certain stimuli such as pH, temperature, magnetic field and electric influence. In addition to that, some of them are responsive to multiple stimuli that cause a physical or chemical change in its arrangement [198, 199].

pH-responsive: Potential targeted sites such as tissues and organs differ in their physiological pH value, which implies a lot of interest for research in this stimulus for triggering and modulating drug release. Ideally, pH-responsive electrospun nanofibers should be designed and developed in a way that makes them able to release the drug at a certain pH close to that caused by a condition or a disease in the biological surroundings [211, 212].

Nanofiber drug release is expected to be reduced or completely stopped whenever the condition is improved and the microenvironment pH shifts back to the physiological value. Most polymers used for this purpose are biocompatible and biodegradable containing carboxylic acids or amine groups, which undergo protonation and deprotonation as well as changes in size, shape, and hydrophobicity causing the drug release mostly through a swelling mechanism [213]. Although pH-responsive electrospun nanofibers have been found useful for many drugs, they are not suitable for protein delivery as pH variations can cause denaturation of these molecules [214].

Thermo-responsive: These systems are composed of polymers that suffer solubility modifications based on the lower critical solution temperature (LCST) (*i.e.* the temperature at which there is a balance in the competition established by hydrophilic and hydrophobic areas of the polymer chain structure) that is usually close to normothermia (37 °C). Thus, below LCST the polymer is completely miscible, but above that parameter, it is only partially due to a reversible phase transition from a hydrophilic form to a dehydrated or hydrophobic state. The aforementioned transition is an entropy-driven process caused by the release of water molecules upon heating [215]. The polymers used for the development of thermo-responsive smart electrospun nanofibers swell below their LCST, which reduces drug release at normothermia because they retain the loaded hydrophobic drug. On the other hand, a small increase in temperature (*e.g.* a locally heated tumor) enhances the release because of nanofibers shrinking, caused by the polymer conversion into a hydrophobic form [216-218].

Magnetic field-responsive: Magnetic field stimulus provides advantages compared to the other options since it is well known that it does not affect tissues and its penetration in the body is greater than heat or light, causing neither cell death nor DNA damage [219, 220]. The magnetic properties allow pharmaceutical nanoparticles to be delivered to a specific site due to the influence of a magnetic field [221]. For the development of these delivery systems, it is necessary to incorporate superparamagnetic iron oxide nanoparticles (SPIONs) with high biocompatibility and low cytotoxicity like Fe_3_O_4_ (magnetite) or Fe_2_O_3_ (maghemite) into the polymers or polymer solution used during the electrospinning process [222, 223]. Magnetite and maghemite display superparamagnetism when the diameter of the nanoparticles is smaller than 20 nm, which results in a non-continuing magnetic interaction upon the removal of an external magnetic field [221].

A common developing strategy involves coating the surface of SPIONs with polymers or encapsulating them in a biodegradable matrix to form a nanocomposite known as magnetic responsive polymer composites (MRPCs) [224]. The formation of MRPCs improves not only SPIONs solubility and biocompatibility but also their surface properties for *in vivo* administration, conferring all of the superparamagnetic related properties to the entire nanocomposite [224]. Magnetically active polymers react to an external magnetic field and exhibit rheological changes or experience mechanical stress. Polymer’s magneto-elastic properties can be used to control deformations such as stretching, contraction, and also the movement of MRPCs [225, 226].

Electric field-responsive: Electrospun nanofibers constituted by electric-responsive polymers experience changes in their volume and swelling mechanism when exposed to an electric field. Electrospinning of ion-doped conducting polymers and polymer composites or coatings has been applied for manufacturing nanofibers intended for electric field-responsive drug delivery system [227-229].

Multi stimuli-responsive: This kind of system represents a new opportunity for electrospun nanofibers with enhanced performance under the influence of two or more signals. Thus, in the case of multiple drug-loaded nanofibers, the release of each drug can respond to a different stimulus, according to the respective polymeric matrix and the manufacturing electrospinning technique. In addition to that, drug release can be triggered at the same time or sequentially, due to the influence of combining multiple stimuli [230, 231].

## Concluding remarks

Nanotechnological development through electrospinning is giving the world the opportunity of overcoming the obstacles presented during the design, formulation, and manufacturing of pharmaceutical, biomedical and bioengineering applications. Currently, electrospun nanofibers are at the forefront of nanotechnology, providing the ability to control and manipulate the properties of the obtained nanostructures, which exhibit improved *in vitro* dissolution performance compared to the conventional solid dispersion systems. Their most important property is related to their large surface area to volume ratio, which is responsible for the optimized performance in combination with particle size reduction and amorphization of the API. Therefore, electrospinning as a formulation strategy that makes use of electrostatic and mechanical forces to spin fibers, is being positioned as a novel and useful method for local and systemic drug delivery of poorly water-soluble drugs. Although the potential benefits from this technique are known, there is still work to do for overcoming safety and scale-up challenges.

## Figures and Tables

**Table 1.** Electrospun smart nanofibers applications.

Smart System	Polymers	Drug	Application	Reference
**pH-responsive**	Eudragit S100, lecithin	Diclofenac sodium	Oral-colon targeted drug delivery.	[200]
	Eudragit L100	Alkaline phosphatase	Peroral delivery of proteins.	[201]
	Eudragit S, Eudragit RS	Indomethacin	Colonic drug delivery.	[202]
**Thermo-responsive**	Poly(*N*-vinylcaprolactam-*co*-methacrylic acid)	Ketoprofen	Extended drug release.	[203]
	Poly(di(ethylene glycol) methyl ether methacrylate), Ethyl cellulose	Ketoprofen	Extended drug release.	[204]
**Magnetic field-responsive**	Poly (d,l-lactide-co-glycolide)	Bortezomib	Implantable device for endoscopic hyperthermia treatment and tumor-triggered controlled drug release.	[205]
	Poly(ε-caprolactone)	Ketoconazole	Slow sustained release for fungal infections.	[206]
**Electrical field-responsive**	Graphene, Poly(ε-caprolactone), Gelatin	Tetracycline hydrochloride	Neural tissue engineering and drug delivery.	[207]
	Poly(ε-caprolactone)	Curcumin	Delivery through programmable electrical devices.	[208]
**Multi stimuli-responsive**	Poly(N-vinylcaprolactam), Ethyl cellulose, Eudragit L100	Ketoprofen	Dual temperature and pH-responsive delivery in lower intestinal tract.	[209]
	Poly-N-isopropylacrylamide, chitosan	Curcumin	Dual temperature and pH-responsive for cancer targeting.	[210]
